# Detecting user experience issues from mHealth apps that support stroke caregiver needs: an analysis of user reviews

**DOI:** 10.3389/fpubh.2023.1027667

**Published:** 2023-05-25

**Authors:** Elton H. Lobo, Mohamed Abdelrazek, Anne Frølich, Lene J. Rasmussen, Patricia M. Livingston, Sheikh Mohammed Shariful Islam, Finn Kensing, John Grundy

**Affiliations:** ^1^School of Information Technology, Deakin University, Geelong, VIC, Australia; ^2^Department of Public Health, University of Copenhagen, Copenhagen, Denmark; ^3^Innovation and Research Center for Multimorbidity, Slagelse Hospital, Slagelse, Denmark; ^4^Department of Cellular and Molecular Medicine, University of Copenhagen, Copenhagen, Denmark; ^5^Center for Healthy Aging, University of Copenhagen, Copenhagen, Denmark; ^6^Faculty of Health, Deakin University, Geelong, VIC, Australia; ^7^Institute for Physical Activity and Nutrition (IPAN), Deakin University, Geelong, VIC, Australia; ^8^Department of Computer Science, University of Copenhagen, Copenhagen, Denmark; ^9^Faculty of Information Technology, Monash University, Clayton, VIC, Australia

**Keywords:** mHealth, APP, stroke, caregiver, user experience, needs, design

## Abstract

**Background:**

Existing research has demonstrated the potential of mHealth apps in improving the caregiving outcomes of stroke. Since most of the apps were published in commercially available app stores without explaining their design and evaluation processes, it is necessary to identify the user experience issues to promote long-term adherence and usage.

**Objective:**

The purpose of this study was to utilize published user reviews of commercially available apps to determine the user experience issues to guide future app development in stroke caregiving.

**Methods:**

User reviews were extracted from the previously identified 46 apps that support stroke caregiving needs using a python-scraper. The reviews were pre-processed and filtered using python scripts to consider English reviews that described issues faced by the user. The final corpus was categorized based on TF-IDF vectorization and k-means clustering technique, and the issues extracted from the various topics were classified based on the seven dimensions of user experience to highlight factors that may affect the usage of the app.

**Results:**

A total of 117,364 were extracted from the two app stores. After filtration, 13,368 reviews were included and classified based on the user experience dimensions. Findings highlight critical issues that affect the usability, usefulness, desirability, findability, accessibility, credibility, and value of the app that contribute to decreased satisfaction and increased frustration.

**Conclusion:**

The study identified several user experience issues due to the inability of the app developers to understand the needs of the user. Further, the study describes the inclusion of a participatory design approach to promote an improved understanding of user needs; therefore, limiting any issues and ensuring continued use.

## 1. Introduction

Stroke caregiving is often associated with persistent psychological distress leading to depression, decreased life satisfaction, and reduced quality of life (1). The impact of stroke caregiving is due to the sudden onset of the disease that requires the caregiver to adjust to a new role with little to no preparation (2) resulting in the caregiver feeling disconnected, isolated, and distant from the recovery process (3).

Technological interventions such as telemedicine, mHealth and so on have in the past highlighted numerous benefits in the healthcare environment to enable caregivers to easily access valuable resources and participate in a variety of activities using their devices (4). These interventions allow the caregiver to ask questions or manage the survivors’ needs at any given place or time (5). Hence, ensuring they feel prepared to manage the disease throughout the disease trajectory (6). In addition to ensuring that the caregiver feels prepared (7, 8), technology in stroke caregiving has the potential to reduce caregiver burden (6, 9), improve caregiver health status (6, 9–12), ensure better healthcare utilization (13), and enhance caregiver self-efficacy and esteem (9, 11, 14).

For over a decade, the number of people using mobile or other portable devices has increased exponentially (15) as a means to communicate with one another and access information at any place or time (16). In 2020, it was estimated that more than 85% of Americans own a smartphone, which is expected to rise in the coming few years (17). Consequently, there has been a significant rise in the development of mobile health apps (18) to address critical healthcare delivery issues through education/awareness, improved risk factor control, efficient screening procedures, and sustainable health system cost reductions (19). The benefits of mHealth apps in healthcare are promising, with research highlighting enhanced support for families caring for their loved ones, improved symptom management, and decreased hospital visits for the survivor (20).

Despite the potential benefits of mHealth applications, the overall adherence of these technologies is relatively low, with most end-users withdrawing from the application within 2 weeks of download (21). For many years, there have been many techniques to measure factors that may contribute to the lack of adherence, including; usability, efficiency, effectiveness, learnability, usefulness etc. In recent times, user experience have been considered by several authors as a recognized standard (22) due to its ability to achieve user satisfaction by focusing on hedonic and pragmatic goals (23).

This study, therefore, aims toward analyzing and evaluating the user reviews of apps that support stroke caregivers healthcare needs (24) based on seven user experience dimensions (25). The results of this analysis can potentially help mobile app developer’s researchers to understand the factors that affect long-term adherence and usage in stroke caregiving technology.

## 2. Methods

### 2.1. App identification

A search strategy was developed based on a previous study (26) to identify apps related to stroke and caregiver. An electronic search was conducted between December 2022 and January 2023 of two app stores (i.e., Google Play Store and Apple App Store) and one commercial mobile repository (i.e., 42 matters). The search involved individual and Boolean searches of stroke and caregiver related MeSH terms identified through PubMed to ensure comprehensiveness.

Apps identified through the search were extracted and stored in a Microsoft Excel spreadsheet, where duplicates within the same platform (such as Android and iOS respectively) were identified and removed. Further, the apps available for different platforms were combined to a single row within the spreadsheet to prevent duplication. The apps were initially screened based on their published meta-data using a well-defined selection criterion (Table 1). After screening, the description of potentially relevant apps was independently reviewed by two authors to determine eligibility.

**Table 1:** Inclusion and exclusion criteria used in the identification of Apps.

Criteria	Description
Inclusion	Published in English language Can be used in stroke care Consists of a description of the app Ability to address the needs of a stroke caregiver, i.e., information, involvement, self-care and support (24)
Exclusion	Not available in English language Not accessible on the app store website Was not reviewed by the user through comments and ratings Was designed for other chronic conditions Was designed for clinicians and/or other healthcare professionals

### 2.2. Review extraction and pre-processing

The user reviews and ratings from included apps were extracted from the app store pages (i.e., Google Play Store and Apple App store) using a Python-based scraper script and stored in a CSV file.

Prior to analyzing the dataset, the data in the CSV file were pre-processed using multiple python-based toolkits to ensure the system can understand the data. The pre-processing technique utilized in this study includes:

- Dataset cleaning and Unicode normalization: It is crucial to have clean and high-quality datasets for any data processing application. The process of dataset cleaning involves splitting text into individual words and handling punctuations and cases. This process was performed using Python NLTK (or Natural Language Toolkit) script making it ready for machine learning and deep learning algorithms. Further, all characters that do not meet the UTF-8 character list were filtered using a Python-based script. For example, the script filtered Unicode characters such as “å” or “ě” and replaced them with “a” and “e,” respectively.
- Stop word removal: Stop words are a list of the most commonly used words that do not have solid semantic properties but are required in a language for communicating information. These words include “the,” “a,” “in,” “and,” “this” and so on (27). The stop words were removed using a stored list present in the Python NLTK to decrease the size of the dataset while reducing the time to train the system and improve the performance during classification.
- Lemmatization: Lemmatization is the process of different grouping words together with a similar meaning to be analyzed as a single item. For example, the term good or better have the same meaning but are represented differently. A Python NLTK script was implemented along with the WordNet Word repository to identify words in the dataset with similar meaning using operations such as tokenizing, classification, stemming, tagging, parsing, and semantic reasoning to ensure greater accuracy.

### 2.3. Review filtration

Positive reviews were excluded from this study as the primary goal was to identify user experience issues present in the app. To determine all the positive, neutral, and negative reviews, sentiment analysis was conducted. Sentiment analysis is a type of text classification that relies on natural language processing, data mining, machine learning, information retrieval, and other processes to indicate the sentiments user expresses (i.e., positive, neutral, or negative) toward a product or feature (28). To perform a sentiment analysis on the dataset, the output of pre-processed reviews was categorized to determine users’ positive, neutral, and negative opinions using a VADER sentiment library in Python.

The VADER library includes a lexicon and rule-based sentiment analysis tool to score text based on its level of positivity and negativity. The tool incorporates numerous lexicon features related to common sentiment expressions, including Western-style emoticons, sentiment-related acronyms, initialisms, and commonly used slang to determine sentimental values. Further, the tool converts feature candidates into sentiment expressions using a wisdom-of-the-crowd (or WotC) approach (29). The outcomes of the sentiment analysis process would be a sum of all compound score values between the ranges of −1 to +1, where the positive sentiment would be greater than or equal to +0.05, and the negative sentiment would be less than or equal to −0.05. All other compound score values would be denoted as neutral sentiments. The negative and neutral reviews were extracted and stored in a CSV file to analyze user experience issues.

### 2.4. Review analysis

The analysis of the negative and neutral reviews involved two stages to identify the user experience issues of stroke caregiving apps. In the first stage, the negative and neutral reviews were classified in python-language based on a TF-IDF vectorization and k-means clustering technique. The Term Frequency-Inverse Document Frequency (TF-IDF) technique is a process that converts the review into usable vector. It generally involves two concepts, (i) term frequency where the number of occurrences of a specific keyword is identified within the corpus represented in the form of a matrix whose rows include the number of reviews and columns represents the distinct terms in the corpus, and (ii) document frequency in which the number of reviews containing a specific keyword is determined. The inverse document frequency, however, determines the weight of a keyword to determine the occurrence of the keyword within the corpus. The TF-IDF score is expected to increase proportionally when the count of a specific keyword increases within the corpus, and can be calculated based on the product of *tf* and *idf* as given below (30):


$$tfidf\left(t,d,D\right)=tf\left(t,d\right)\times idf(t,D)$$

where *t* denotes the terms; *d* denotes each document; D denotes the collection of documents.

Term Frequency (tf):


$$tf\left(t,d\right)=\frac{Number\;of\;times\;the\;keyword\;t\;appears\;in\;a\;review}{Total\;number\;of\;keywords\;in\;the\;corpus,d}$$

Inverse Document Frequency (idf):


$$idf\left(t,D\right)=\log_{e}\frac{Total\;number\;of\;reviews\;in\;a\;corpus,D}{Number\;of\;reviews\;with\;keyword\;t\;in\;it}$$

The vectorized representation of the reviews is classified based on *k*-means clustering approach. *k*-means is an unsupervised learning algorithm that classifies the reviews into a certain number of clusters. The main idea is to define *k*-centroids for each cluster, and represent the vectorized data to the nearest centroid. The algorithm calculates the average position of the points based on the respective centroid and updates the position to determine the group to which each review belongs (31). The clusters are finalized when all points are at a minimum distance from their respective centroid, which are exported in the form of a CSV file.

During the second stage, two reviewers independently identified key issues within the apps based on the clusters represented within the CSV file. These issues were independently classified by the same two reviewers using NVivo 12 following the seven dimensions of user experience design, i.e., usable, useful, desirable, findable, accessible, credible, and valuable as shown in Table 2. Any discrepencies in the identification and classification of issues were discussed by all authors until a general consensus was achieved.

**Table 2:** User experience dimensions as described by Morville (25).

Dimension	Definition
Usable	The system is easy to use and understand to achieve a desired goal effectively, efficiently and satisfactorily
Useful	The system needs to be useful and address the needs and wants of the user
Desirable	The visual esthetics of the system needs to be attractive and easy to translate
Findable	The functions of the systems needs to be findable and easy to navigate
Accessible	The system needs to be designed in such a way that users with disabilities can have the same user experience as others
Credible	The developer or company providing the system needs to be trustworthy
Valuable	The system needs to solve problems and deliver a return on investment

## 3. Results

**Figure 1:**
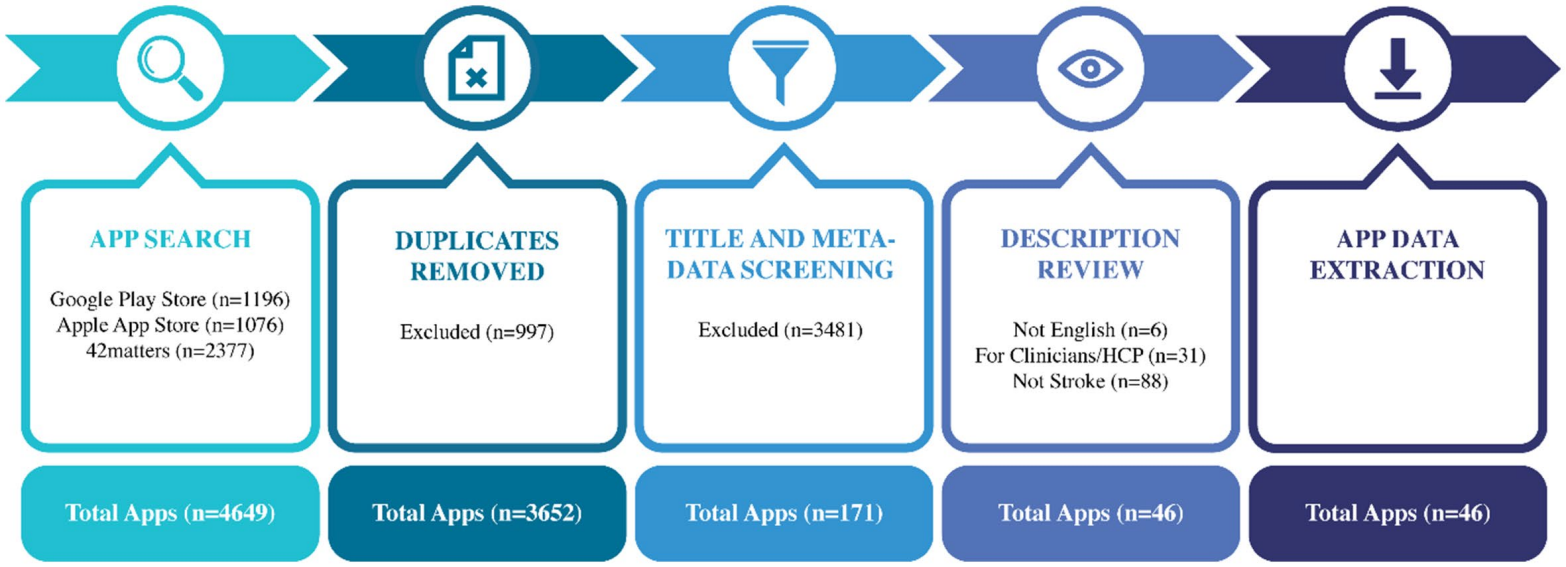
App identification process.

The initial search yielded a total of 4,649 apps from various app stores (i.e., Google Play Store and Apple App Store) and app repository (42 matters). After removing duplicates, a total of 3,652 apps were screed based on their titles and meta-data. The descriptions of 171 apps were reviewed, of which 46 were included in this study. Figure 1 represents the app identification process used in the study.

**Figure 2:**
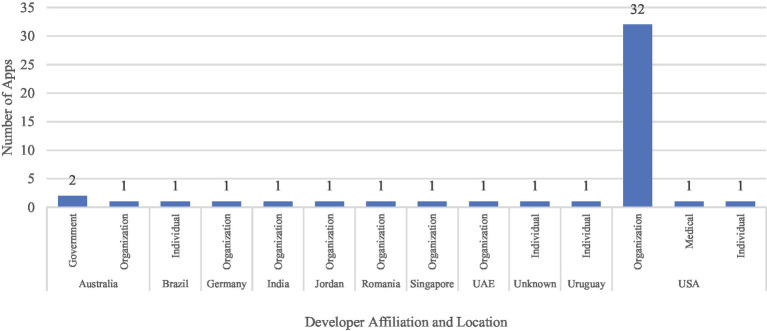
Distribution of Apps based on the developer affiliation and location.

**Figure 3:**
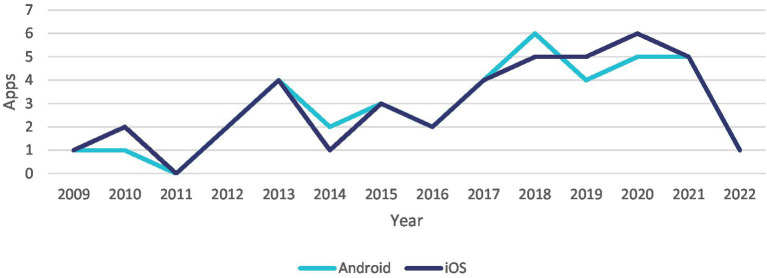
Distribution of Apps based on the publish year and platform.

A majority of the apps were developed by organizations (*n* = 39) generally located in the United States (*n* = 32). In addition, only two apps were developed by organizations funded by the government in Australia, and one app was developed by a healthcare professional in the United States as shown in Figure 2. The included apps were developed between 2009 and 2022 as illustrated in Figure 3 in both Android (*n* = 40) and iOS (*n* = 41) platforms. The features of these apps aimed at supporting a myriad of health needs, which can be classified into four critical needs especially in stroke caregiving, i.e., information (*n* = 15), involvement in care (*n* = 32), self-care (*n* = 4), and support (*n* = 23). Overall, the apps have a positive user rating with an average of 4.2 on a scale of 1–5, where 1 is the lowest rating and 5 is the highest rating. Supplementary Table S1 in the Supplementary material consists of the characteristics of apps included in this study.

### 3.1. Filtration of user reviews

**Figure 4:**
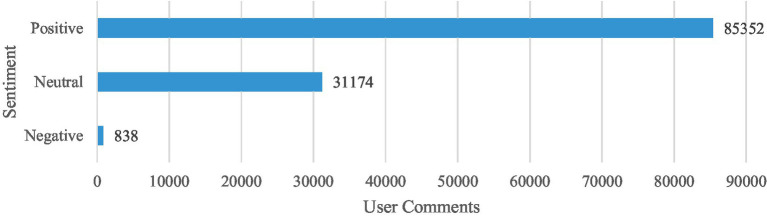
Distribution of App comments based on its individual sentiments.

The app meta-data extracted account for a total of 422,647 user reviews from the 46 apps included in the study. Out of 422,647 user reviews available, the python-based scraper extracted only 117,364 due to the limitations of both app stores (i.e., Google Play Store and Apple App Store), which formed the initial dataset for this study. The dataset was categorized based on user sentiments into positive (85,352/117,364), neutral (31,174/117,364), and negative (838/117,364) user reviews as illustrated in Figure 4. Positive reviews (85,352/117,364) and reviews not published in English (388/117,364) were excluded from the study forming a corpus of 31,624 user reviews that were classified based on the user experience dimensions described in Table 2.

### 3.2. Characteristics of user reviews

The corpus of 31,624 user reviews represents the user experience issues of 38 out of 46 apps as described in the Supplementary Table S1 in the Supplementary material. These user reviews consists of 11,360 unique users and 18,256 users with deleted accounts. Out of the 11,360 unique users, 9,385 users have only posted a single review, 1,910 users have posted two reviews, 46 users have posted three reviews, 17 users have posted four reviews, one user had posted five reviews, and one user had posted six reviews. These user reviews were posted in one or more apps.

Reviews posted by users with deleted accounts were excluded to ensure the findings are more trustworthy. Hence, 13,368 users reviews formed the final corpus that was analyzed in this study.

### 3.3. Analysis of the user reviews

**Figure 5:**
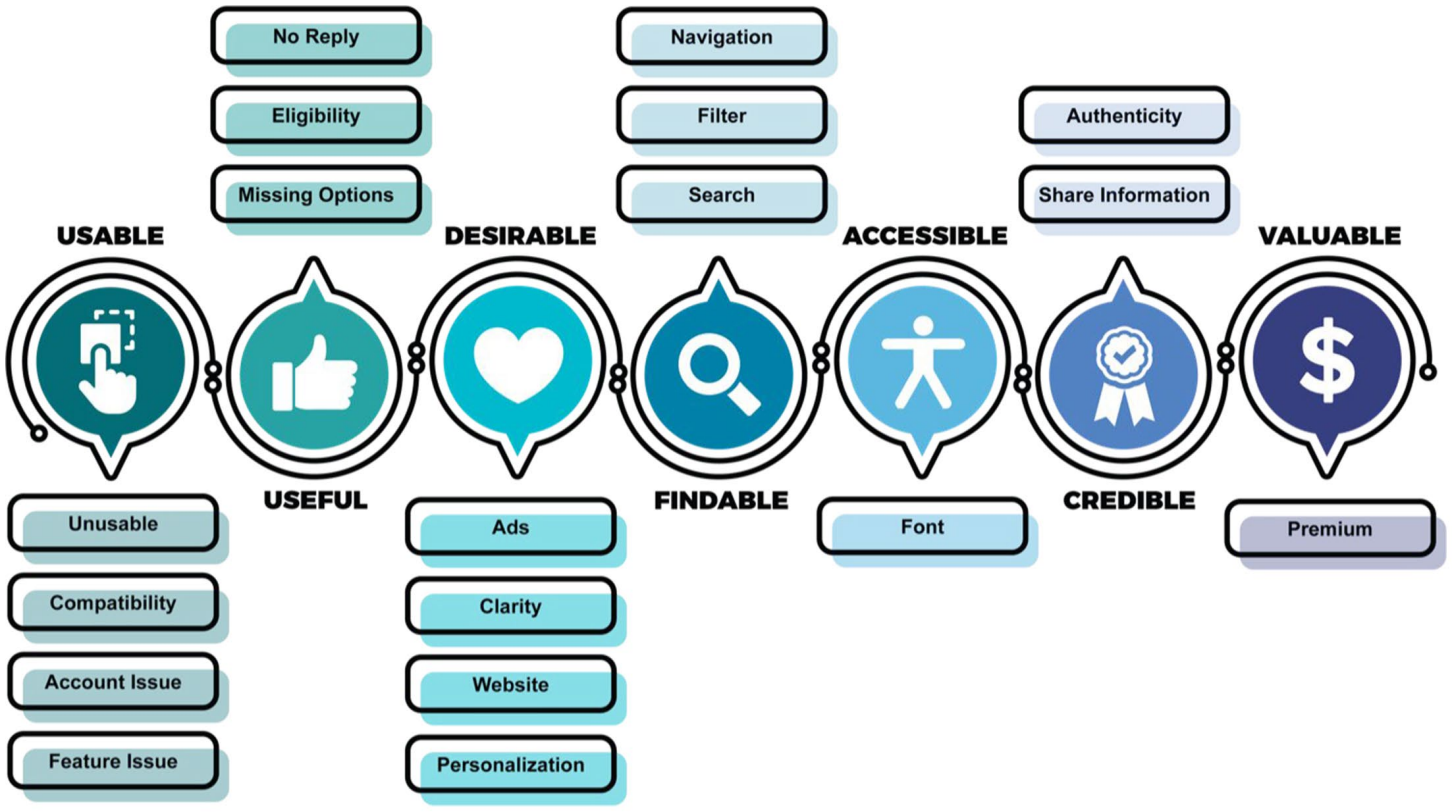
User experience issues.

Twenty-six clusters were identified based on the TF-IDF vectorization and *k*-means clustering technique as illustrated in Supplementary Table S2 in the Supplementary material. Each topic represents several issues within the app as shown in Figure 5 that were classified based on the dimensions in Table 2 to determine factors that affect overall user experience.

#### 3.3.1. Dimension #1: usable

The first dimension, usability, consisted of four critical issues (unusable, compatibility, account issue, and feature issue) as described in several app reviews. The first issue described by the user was that the app was unusable due to constant crashes or freezing. In some cases, the app would also be slow to use or did not work which contributed to frustration. The second issue is with regard to compatibility, which is the inability of the app to work of a myriad of mobile devices irrespective of the platform. The third issue is related to the user account, where users described being unable to login or change password. In most cases, the users would receive an error, which is unclear and leads to confusion. The users, who were able to access their accounts, felt that the login process was complex and needed to be simplified, with one suggestion being the inclusion of a fingerprint login feature to reduce the number of steps. The fourth issue that is related to the app features was the most discussed that affected the overall user experience. Topics such as issues with (i) the reminder providing alerts at right times or being audible, (ii) missing notifications/alerts, (iii) location/GPS issues, (iv) internet or server connection issues, (v) customization of features such as alert volume and tunes, (vi) unresponsive buttons and scroll, (vii) effectiveness of the app in managing medication refills, and (viii) data upload were discussed by users within the app reviews. Users described the need for developers to fix these issues to improve their experience. Moreover, users described that the app widget, scheduling, and interface was difficult to use and outdated, with suggestions to include tutorials or help options to assist the user.

#### 3.3.2. Dimension #2: useful

The second dimension, usefulness, was affected by three issues. The first issue is with the eligibility of the app. One user review described that the app was only available to adults, which resulted in it being uninstalled. Another issue narrated by the user includes the inability of the app to address all the needs of the user such as a complete medication list or missing features. Missing features reported in the reviews include ability to customize notifications, ability to log notes, features to edit and manage medications, ability to search and filter data, ability to import, export and print data, and ability to synchronize with other calendars or devices. The final issue faced by the user using an app to engage assistance from a formal caregiver was the lack of reply they would get to their message.

#### 3.3.3. Dimension #3: desirable

The third dimension, desirability, consists of four issues related to the interface of the app. The critical issue that affects desirability is the inclusion of several advertisements (or ads) within the app. Users felt frustrated and annoyed with the number of ads they had to view to use the functionality of the app. Other issues included the lack of clarity and intuitiveness in the app interface making it difficult to understand, and the lack of personalization within the app to customize the app interface. For example, an app review described the inclusion of a dark mode feature. In addition, users reported issues with the website interface when accessing the app through other devices.

#### 3.3.4. Dimension #4: findable

The fourth dimension, findable, consists of three issues with regard to the search, filter, and navigation of the app. Users described not being able to search or filter through their medication list making it difficult to perform their activities. The app reviews suggested including proper search options to find the required data. In addition, users found the navigation of the app extremely hard and confusing, with users suggesting the enhancement of menus to improve ease of use.

#### 3.3.5. Dimension #5: accessible

The fifth dimension, accessibility, consists of one main issue, which is the font. Users reported having issues with the font size and color within the app that affected their ability to engage with the content provided.

#### 3.3.6. Dimension #6: credible

The sixth dimension, credibility, consists of two main issues that leads to users uninstalling the app from their mobile devices. The first issue is authenticity of the app as users felt they were being scammed into paying for the features within the app. Moreover, users that contacted customer care services for assistance did not receive a reply, which increased their fears. Another issue faced was the reluctance of users in sharing their personal information with the app due to suspicions related to data privacy and security.

#### 3.3.7. Dimension #7: valuable

The seventh dimension, valuable, consists of one critical issue that is the reluctance of users to pay from premium version. This is because users were looking for cheaper options and/or did not feel the app was worth upgrading to unlock the premium features, which resulted in the app being uninstalled by the user. One suggestion uncovered from the app reviews describes the possibility of including more options within the free version to make the user rethink their decision to uninstall the app.

## 4. Discussion

### 4.1. Principal findings

The purpose of this study was to identify the user experience issues faced by users of apps that can assist with the needs of stroke caregiving. The study included 46 apps on Android and iOS platforms, with over 422,647 user comments and a high average user rating (i.e., 4.2) on a scale of 1–5. On extraction of user comments, however, only 117,364 user comments were extracted due to app store restrictions with similar issues faced in the study by Maalej and Nabil (32). After filtration, 31,624 neutral and negative user comments in 39 apps were classified based on the seven dimensions of user experience to determine potential issues.

Understanding factors such as user experience is critical as it creates a positive relationship between the product, the user, and the organization (33) while also ensuring the system’s long-term success (21, 34). This relationship was evident in this study, where several users reported the need to withdraw from the app due to issues with the app user experience. However, these issues could be expected as most apps were published by non-educational/medical organizations with limited evaluations.

Beyond the user experience issues, several users described the app’s inability to support their needs, which contributed to their dissatisfaction with the app. Moreover, Torous, Andersson, Bertagnoli, Christensen, Cuijpers, Firth, Haim, Hsin, Hollis, Lewis, Mohr, Pratap, Roux, Sherrill, and Arean (21) suggests that the inability to align the app functionalities with the preferences and goals of the intended users may lead to a lack of adherence that may eventually influence the app usability. For example, one user mentioned that the paid app prices are incredibly high, especially for people assisting those with special needs that would require lifestyle adjustment and unavoidable financial responsibilities, which may affect their ability to engage with the app. Another user mentioned the need to include other useful aspects such as medication information, photo and notes in a medication management app to allow better support.

The design of any commercially available mHealth app ultimately depends upon the uptake and success of the app, which is found to be linked with the need to design the system based on user preferences and goals. Moreover, the app needs to function in a way to promote improved user experience. Hence, developers need to consider an approach that can understand the needs, engage the end-user and priorities the requirements to ensure effective outcomes. User-centered design is one such approach.

The user-centered design had been endorsed by the World Health Organization (WHO) as an effective approach to ensure improved outcomes in terms of usability and functionality (35). This approach provides the better inclusion of target end-users during the design and development of the app based on a clear understanding of the processes involved in the planning of care and recovery (36). Furthermore, if methods such as participatory design are implemented, it can create meaningful, actionable, and feasible strategies (37).

Participatory design has been used to align the concerns of users with health technologies (37). This is because the traditional design approaches fail to engage users in the design process, which eventually compromises the commercial opportunity and interactional experience of the users (38). Kushniruk and Nøhr (39) reported benefits of user involvement, particularly in participatory design, including (i) improving system quality as a result of more accurate understanding of user needs and preferences, (ii) greater likelihood of inclusion of features that are required by the user, (iii) higher levels of user acceptance as the system was developed based on user input, (iv) improved understanding of the usage issues, training needs, and user engagement, and (v) higher level of participation in the user decision making processes. Hence, making it an ideal approach in the design of mHealth technologies, especially in stroke caregiving.

Despite the numerous user experience issues, it is essential to note the high level of satisfaction among the user of the extracted app, with an average rating of 4.2 on a scale from 1 to 5 (26). Some users have discussed the presence of fake reviews in their user comments. For example, one user mentioned “many five star ratings” without any “meaningful” comments. In contrast, another user discussed the feeling that most fake positive reviews were posted by the developer for an “obviously subpar product.” The suspicion for fake reviews can be further supported with a large number of reviews posted from deleted accounts as seen in this study and the rise in the illegal market for fake reviews to help app developers improve their rankings and ratings (40). These fake reviews have not only misled many customers into making poor decisions but also affect the users’ trust in online reviews (41) as seen in several published user comments.

### 4.2. Strengths and limitations

This study has several notable strengths. The primary being the novelty. To the best of our knowledge, the analysis of user feedback in apps that support the needs of stroke caregivers has not been addressed. As a result, addressing a key gap in the literature. It also provides a voice to a large sample of users, highlighting their needs and expectations from the app. This feedback can be used to establish support for user inclusion in the design and development processes of the app. Moreover, it can provide future developers with the necessary guidelines in app design. In addition to providing a novelty and providing a voice to app users, the study is comprehensive. It gives a precise classification of user experience issues based on the seven dimensions of user experience design with high interrater reliability for each dimension.

Despite the strengths, the study includes a few limitations. First, the comments extracted were less than 28% than those published due to app store limitations. Another downside is the sensitivity of sentiment analysis used in this study. While sentiment analysis has been successfully applied to numerous different applications to understand user opinion, a few neutral or negative reviews may have been falsely classified as positive, which would have resulted in its exclusion from this study. The inclusion of more comments may have painted a different picture of the user experience issues and may have uncovered other problems within the app. Furthermore, the findings of this research only provides a high-level view of possible issues that the topic encapsulates, which may be different if a thematic analysis of reviews were considered.

## 5. Conclusion

The study explores the user experiences issues of apps that support the needs of stroke caregivers in their daily activities and considerations for future app development. The implication is to inform the development of apps by considering users using user-centered design approaches such as participatory design. Most apps have demonstrated a lack of understanding of user needs that contribute to user experience issues. Therefore, resulting in the lack of adherence and affecting user satisfaction. Hence, the collaboration with necessary stakeholders could contribute to the design of an app that is meaningful, actionable and feasible to the user preferences and goals.

## Data availability statement

The original contributions presented in the study are included in the article/Supplementary material, further inquiries can be directed to the corresponding author.

## Author contributions

EL, MA, and JG designed and conceptualized the study. EL coordinated the classification process between the two coders and analyzed the data under the supervision of MA. Further, EL drafted the manuscript based on the findings, which were reviewed, modified, and approved by MA, AF, LR, PL, SI, FK, and JG involved in the study. All authors contributed to the article and approved the submitted version.

## Funding

This study was supported through doctoral scholarships from the School of Information Technology, Deakin University, and the Department of Public Health, University of Copenhagen. Further, JG was supported by ARC Laureate Fellowship FL190100035.

## Conflict of interest

The authors declare that the research was conducted in the absence of any commercial or financial relationships that could be construed as a potential conflict of interest.

## Publisher’s note

All claims expressed in this article are solely those of the authors and do not necessarily represent those of their affiliated organizations, or those of the publisher, the editors and the reviewers. Any product that may be evaluated in this article, or claim that may be made by its manufacturer, is not guaranteed or endorsed by the publisher.

## Abbreviations

mHealth: Mobile health
NLTK: Natural language toolkit
LDA: Latent dirichlet allocation
CSV: Comma separated values
App: Application
TF-IDF: Term frequency-inverse document frequency
